# A new trematopid from the lower Permian of Oklahoma and new insights into the genus *Acheloma*

**DOI:** 10.1371/journal.pone.0309393

**Published:** 2024-10-17

**Authors:** Adrian F. Osterling Arias, Ethan D. Mooney, Joseph J. Bevitt, Robert R. Reisz

**Affiliations:** 1 Department of Biology, University of Toronto at Mississauga, Mississauga, Ontario, Canada; 2 International Center of Future Science, Dinosaur Evolution Research Center, Jilin University, Changchun, Jilin, Peoples Republic of China; 3 Australian Nuclear and Science Technology Organization (ANSTO), Australian Centre for Neutron Scattering, Lucas Heights, Sydney, New South Wales, Australia; Chinese Academy of Sciences, CHINA

## Abstract

Modern-day terrestrial amphibians pale in comparison to their monstrous ancient relatives, the Late Carboniferous and Early Permian trematopid temnospondyls. With a skeleton that clearly indicated a terrestrial mode of life and armed with an impressive set of large, recurved marginal dentition and palatal fangs for holding their prey—this group of terrestrial temnospondyls roamed North America and Central Europe as a top predator. Lack of substantial informative fossil material has previously limited our understanding of trematopid diversity and ontogeny. Fortunately, this has improved in the last few decades with the help of exceptional localities like the Early Permian locality Richards Spur. While multiple species of dissorophid temnospondyl have been described from Richards Spur, only one trematopid species has been confidently recognized -*Acheloma dunni*. Here, we report on the presence of a new large, relatively mature trematopid skull from this famous locality, found encased within a limestone rich block composed mostly of skeletal remains from several other taxa. With the help of neutron computed tomography (nCT), a non-invasive method of analyzing internal and external morphologies, this specimen has revealed several features consistent with the genus, *Acheloma*, but distinct from *Acheloma cumminsi* and *Acehloma dunni*. The identification of these new features, in addition to the characters it shares with other *Acheloma* species, not only constitute it as a new species of this genus, but also challenges the notion of having synonymized *Acheloma dunni* with *Acheloma cumminsi*. In this study, we also unveil new anatomical characters that are potentially independent of ontogeny and could therefore help clarify some of the phylogenetic relationships of this fascinating group of Paleozoic terrestrial predators.

## Introduction

Amphibians today pale in comparison to the ferocity and terrible majesty of their ancient relatives, the trematopids. These ancient amphibians were one of the earliest, and only terrestrial tetrapod apex predators of the Dissorophoidea superfamily. The unusual terrestrially-focused morphologies they developed as amphibians makes them a valuable piece in the puzzle for understanding this major chapter in vertebrate evolution, despite still being tied to water for reproductive and breeding purposes much like their extant descendants [1].

Although they generally lacked the heavy dermal armour of dissorophids, trematopids remained as top predators who preyed upon a variety of smaller vertebrates during the early Permian periods of North America [2–11] and Central Europe [1,12,13]. Like many other dissorophoids, trematopids reached their peak diversity during the Early Permian, with some taxa like *Acheloma dunni* [10] and *Acheloma cumminsi* [7] having skulls nearly 22 cm in length, strong postcranial skeletons, and musculature adept for a terrestrial lifestyle [10,14]. These likely ambush predators supported a large, elongated triangular-shaped skull equipped with large palatal fangs and hundreds of tiny denticles meant for holding their prey and feeding on other tetrapods [10,15]. Although the monophyly and synapomorphies of the clade are considered to be well-supported, the interrelationships within the clade remains unsettled largely due to the lack of informative fossil material and a limited understanding of their ontogeny [8,16]. Currently, ten genera and twelve species of trematopids are confidently known, most of which are found across North America in a select few localities (e.g. Oklahoma, USA [10], Texas, USA [7], Kansas, USA [17], New Mexico, USA [5]).

The fossil rich cave deposits near Richards Spur, Oklahoma, have recently yielded a wealth of incredibly preserved material owing to the deposition in fine clays and complex interactions of carbonate rich ground water, oil seep associated hydrocarbons, and cave conditions [18,19]. Although the majority of the preserved fossil material within the cave systems are disarticulated and undistorted, a few specimens remain articulated, thereby allowing great opportunities to study not only trematopids, but also a variety of other early terrestrial tetrapods.

Previously, the sole recognized trematopid at Richards Spur was *Acheloma dunni* [10,18], although disarticulated material has been tentatively assigned to the Texan trematopid *Acheloma cumminsi* [7,20,21]. Recently, the holotype of *A*. *dunni* was declared a junior synonym of *A*. *cumminsi* and a fascinating juvenile trematopid from this site was also interpreted as *A*. *cumminsi* [21]. It is in this context that we present a new exceptionally preserved skull from a new species of trematopid for the purposes of further elucidating on the taxonomic diversity of trematopids at Richards Spur. As such, it is rather interesting that two large predatory trematopids could have occupyied the same community, which speaks to the complex ecology of the Richards Spur community.

## Materials & methods

### Neutron tomography

This study employed the DINGO thermal-neutron imaging instrument at the Australian Nuclear Science and Technology Organisation (ANSTO), Lucas Heights, NSW, Australia [22]. DINGO utilises a quasi-parallel, collimated beam of neutrons generated by the OPAL multipurpose nuclear research reactor and can be configured in either of two collimation (L/D) ratios of 500 or 1000, where L is the neutron aperture-to-sample length and D is the neutron aperture diameter. For this measurement an L/D ratio of 1000 was used to ensure highest available spatial resolution, neutrons were converted to photons using a 200 × 200 × 0.100 mm ZnS/6LiF scintillation screen and photons detected by an Andor IKON-L CCD camera (liquid cooled, 16-bit, 2048 × 2048 pixels) coupled with a 50 mm Carl Zeiss lens, yielding a pixel size of 0.0965 mm. A total of 2881 equiangular radiographs were acquired as the specimen was rotated 360° about its vertical axis, with each radiograph consisting of 2 x 22s exposures [23], for a total scan time of 48h. The raw data were treated in ImageJ v.1.51h to remove salt-and-pepper noise, intensity normalized, and duplicate images averaged using the “Grouped ZProjector” plugin prior to tomographic reconstruction using Octopus Reconstruction v.8.8.

Exposure to neutrons during the procedure of neutron tomography results in temporary radioactivation of all specimens. After 48h exposure to a thermal-neutron flux of 1.15 × 107 n/cm2/s, the immediate post-irradiation contact dose rate of ROMVP 88291 was 72 mSv/h, and it was stored onsite for 3 weeks until residual radioactivity had returned to natural activity levels.

ImageJ (version 1.53a) was used to convert the 16-bit TIFF slices from the above-mentioned scan into 8-bit and improve their contrast. The subsequent image sequences was then rendered using the 3D segmentation software Aviso Lite (version 2020.3) registered to R.R. Reisz at the University of Toronto Mississauga.

### Nomenclatural acts

“The electronic edition of this article conforms to the requirements of the amended International Code of Zoological Nomenclature, and hence the new names contained herein are available under that Code from the electronic edition of this article. This published work and the nomenclatural acts it contains have been registered in ZooBank, the online registration system for the ICZN. The ZooBank LSIDs (Life Science Identifiers) can be resolved and the associated information viewed through any standard web browser by appending the LSID to the prefix “https://zoobank.org/”. The LSIF for this publication is urn:lsid:zoobank.org:pub:AA1FC920-1464-4D36-98B8-E31968374E4B. The electronic edition of this work was published in a journal with an ISSN, and has been archived and is available from the following digital repositories: PubMed Central, LOCKSS.

### Systematic paleontology

    **Temnospondyli Zittel, 1888**

    **Dissorophoidea Bolt, 1969**

    **Xerodromes Schoch and Milner, 2014**

    **Olsoniformes Anderson et al., 2008**

    **Trematopidae Williston, 1910**

    ***Acheloma* Cope, 1882**

    ***Acheloma cryptatheria* sp. nov.**

### Etymology

*cryptatheria* **=** κρυπτός (kruptos) “hidden” + ther-, θήρ or θηρός (thēr), Greek, “beast”.

The species name *cryptatheria* is derived from the Greek *crypta* describing the hidden nature of the skull within a block of numerous other bones, and the Greek *theria* reflects its beastly appearance and role as an apex predator.

### Material

ROMVP 88291, a nearly complete articulated skull within a block of various disarticulated skeletal elements from various other tetrapods. This specimen has undergone CT segmentation and is currently on loan from the vertebrate paleontology collections department at the Royal Ontario Museum, Toronto, ON, Canada.

No permits were required for the described study, which complied with all relevant regulations.

### Locality

The Dolese Brothers limestone quarry, located near Richards Spur, Oklahoma, is a ‘Lagerstätte’ preserving the richest and most diverse assemblage of Paleozoic terrestrial vertebrates. This infilled cave system dates to the early Permian and has more than 40 known taxa, all of which are fully terrestrial tetrapods [18]. Infiltration and permeation of organic remains by action of carbonate rich groundwaters and oil seeped hydrocarbons within a cave setting is attributed to exceptional preservation of largely disarticulated, as well as articulated and semi-articulated skeletal remains, and even soft tissues [18,19].

### Diagnosis

Large trematopid temnospondyl characterized by the following autapomorphy: ectopterygoid with a lateral exposure along its entire length, exceeding the diameter of the orbit, making direct contact with the quadratojugal and excluding the maxilla from contact with the lacrimal and jugal. Currently, the following features appear to only reliably distinguish this large trematopid from other adult members of the genus *Acheloma*: maximum skull width at the level of the pineal foramen; strong presence of anteroposteriorly trending ridge and groove sculpturing of the snout; and a maximum skull width that is greater than the total midline skull length.

### Description

**Fig 1:**
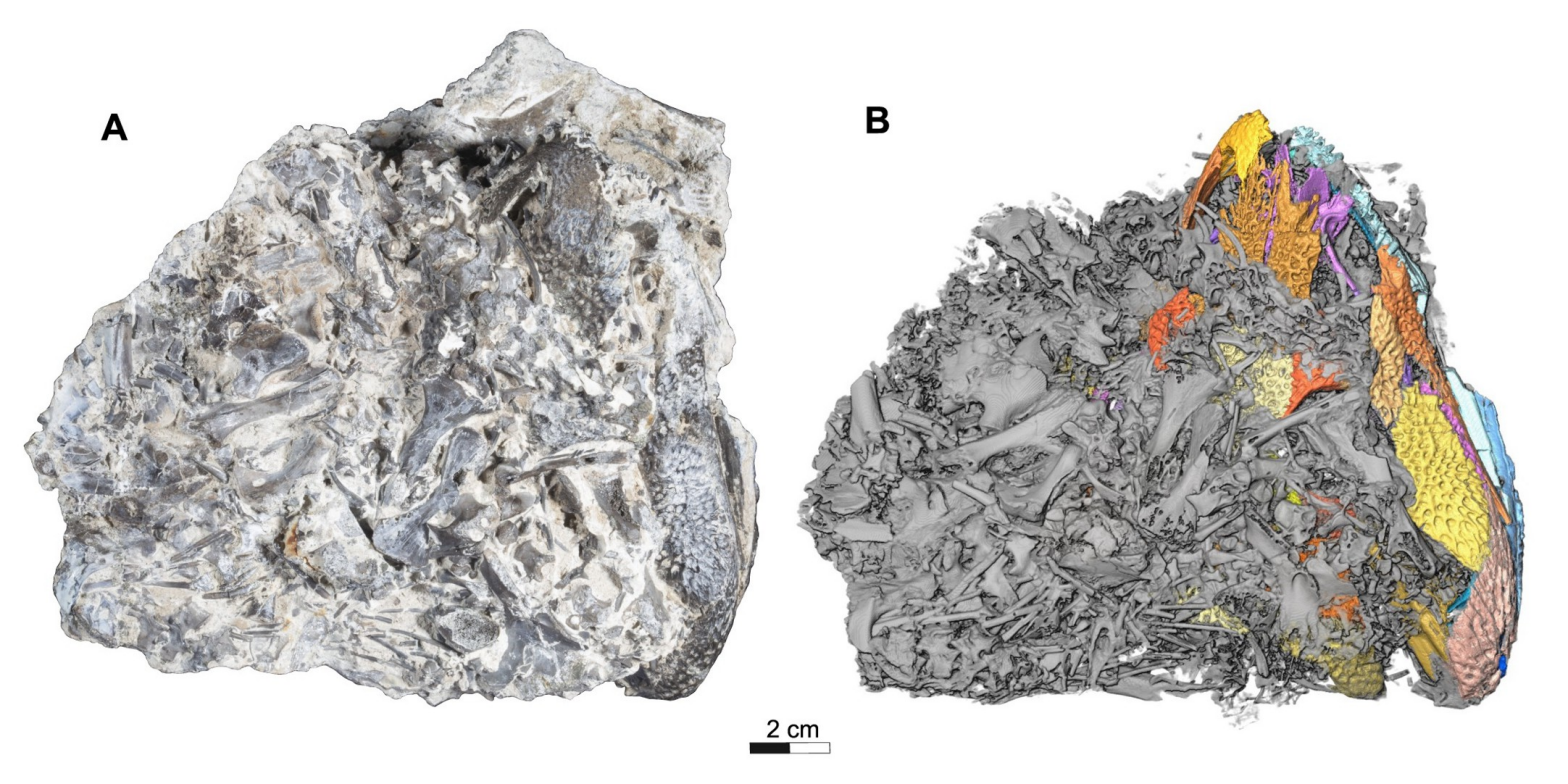
Dorsal views of *Acheloma cryptatheria* (ROMVP 88291) and corresponding grayscale rendering. (A) Photograph and (B) three-dimensional rendering of ROMVP88291 specimen in dorsal view. Note: in grayscale view, the trematopid skull is highlighted in different shades of yellow, brown, purple, and blue that clearly distinguishes it from other unrelated skeletal remains. Scale bar equal to 2 cm.

**Fig 2:**
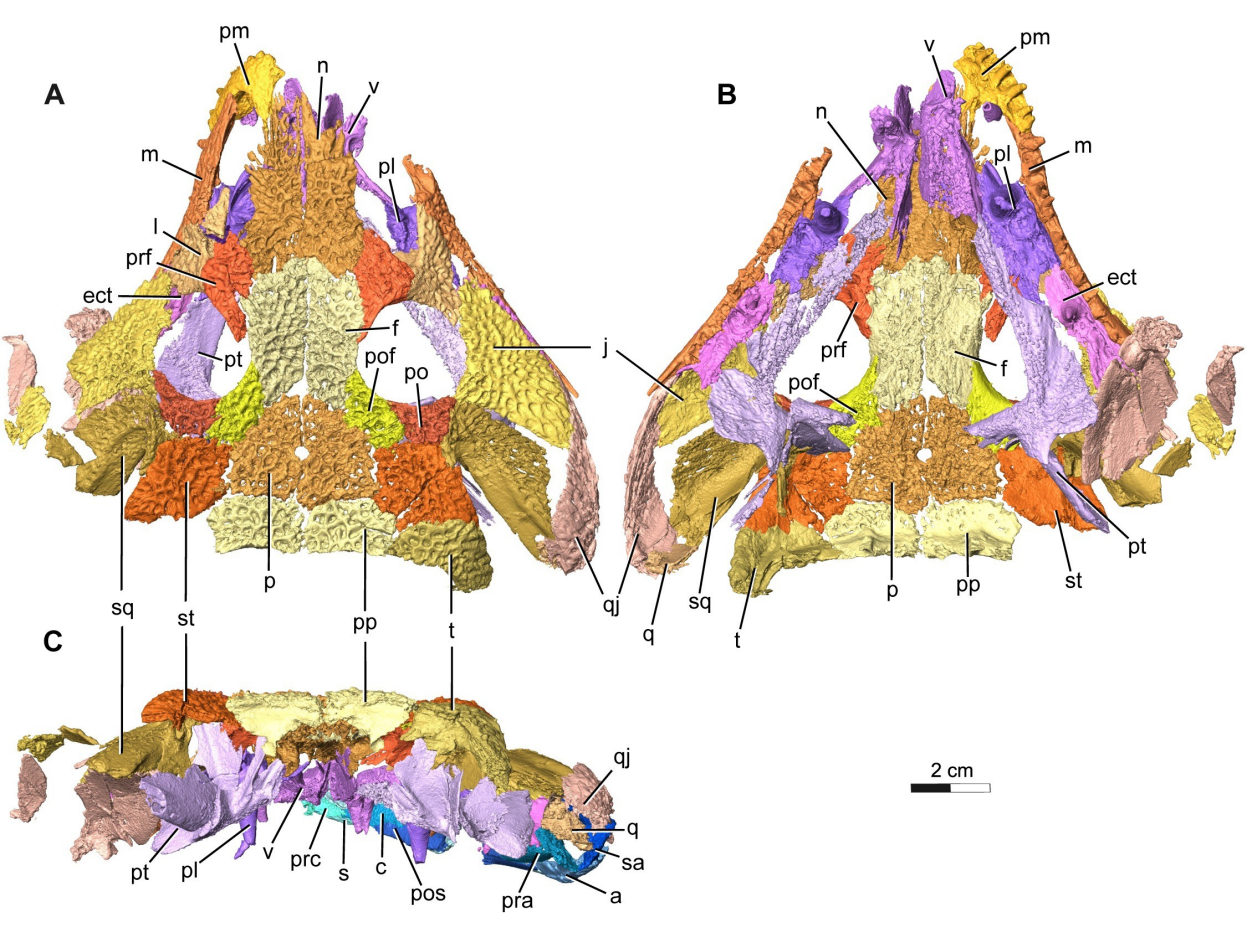
Segmentation rendering of *Acheloma cryptatheria* (ROMVP 88291) in dorsal, ventral and occipital view from CT image sequence. Three-dimensional model of *Acheloma cryptatheria* skull roof and palate in (A) dorsal view, (B) ventral view, and (C) occipital view. Abbreviation: a, angular; c, coronoid; ect, ectopterygoid; f, frontal; j, jugal; l, lacrimal; m, maxilla; n, nasal; pl, palatine; p, parietal; pof, post frontal; po, post orbital; pp, post parietal; pra, prearticular; prc, precoronoid; prf, prefrontal; pm, premaxilla; pt, pterygoid; q, quadrate; qj, quadratojugal; s, splenial; sq, squamosal; st, supratemporal; t, tabular; v, vomer. Scale bar equal to 2 cm.

**Fig 3:**
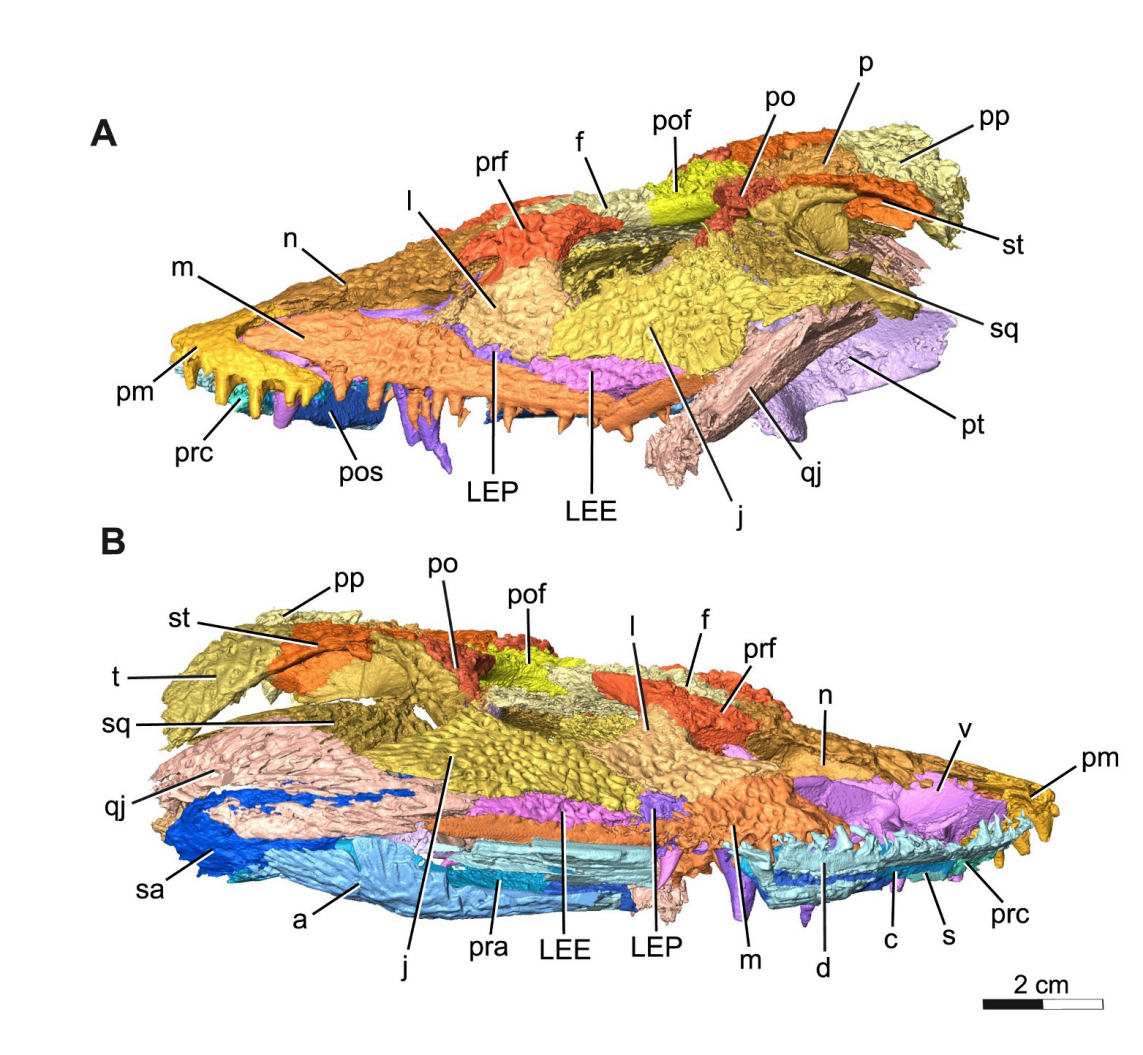
Segmentation rendering of *Acheloma cryptatheria* (ROMVP 88291) in right and left lateral view from CT image sequence. Three-dimensional model of *Acheloma cryptatheria* skull roof, palate and mandible in (A) left lateral view and (B) right lateral view. Abbreviation: a, angular; c, coronoid; d, dentary; f, frontal; j, jugal; l, lacrimal; LEE, lateral exposure of ectopterygoid; LEP, lateral exposure of palatine; m, maxilla; n, nasal; p, parietal; pof, post frontal; po, post orbital; pp, post parietal; pos, postsplenial; prc, precoronoid; prf, prefrontal; pm, premaxilla; pt, pterygoid; qj, quadratojugal; s, splenial; sq, squamosal; st, supratemporal; sa, surangular; t, tabular; v, vomer. Scale bar equal to 2 cm.

The holotype and current only known specimen ROMVP 88291 is typical of trematopids, specifically *Acheloma*, in exhibiting a broad, flat, triangular shaped skull, with characteristic dermal sculpturing [6,8,12,24,25]. A portion of the snout and right mandible of ROMVP 88291 is exposed, while the rest of the articulated skull remains concealed within a block of limestone rich sediment that also contains largely disarticulated skeletal material from a variety of other tetrapods, much of which appears to belong to varanopid *Mesenosaurus* (Fig 1). This nearly complete skull lacks the left mandible, right premaxilla, septomaxillae, articulars, left quadrate, left tabular, and the entire braincase (Figs 2 and 3). Some of the missing elements, like the braincase appear to have been lost prior to, or during deposition, possibly from secondary fluvial transport within the cave system. Other parts of the skull, like elements of the lower jaw were likely lost during blast mining as part of active quarrying operations. While the skull roof is slightly depressed due to limited post-mortem disarticulation, there is no apparent lithostatic distortion of any elements. In our description and comparison, we distinguish between the relatively poorly preserved, somewhat overprepared specimens of *Acheloma cumminsi* [7], and the superbly preserved holotype of *Acheloma dunni* [10]. Although these have been synonymized, we find sufficient differences between them to consider them separately and expand on this topic in the discussion [21].

### Skull roof

**Fig 4:**
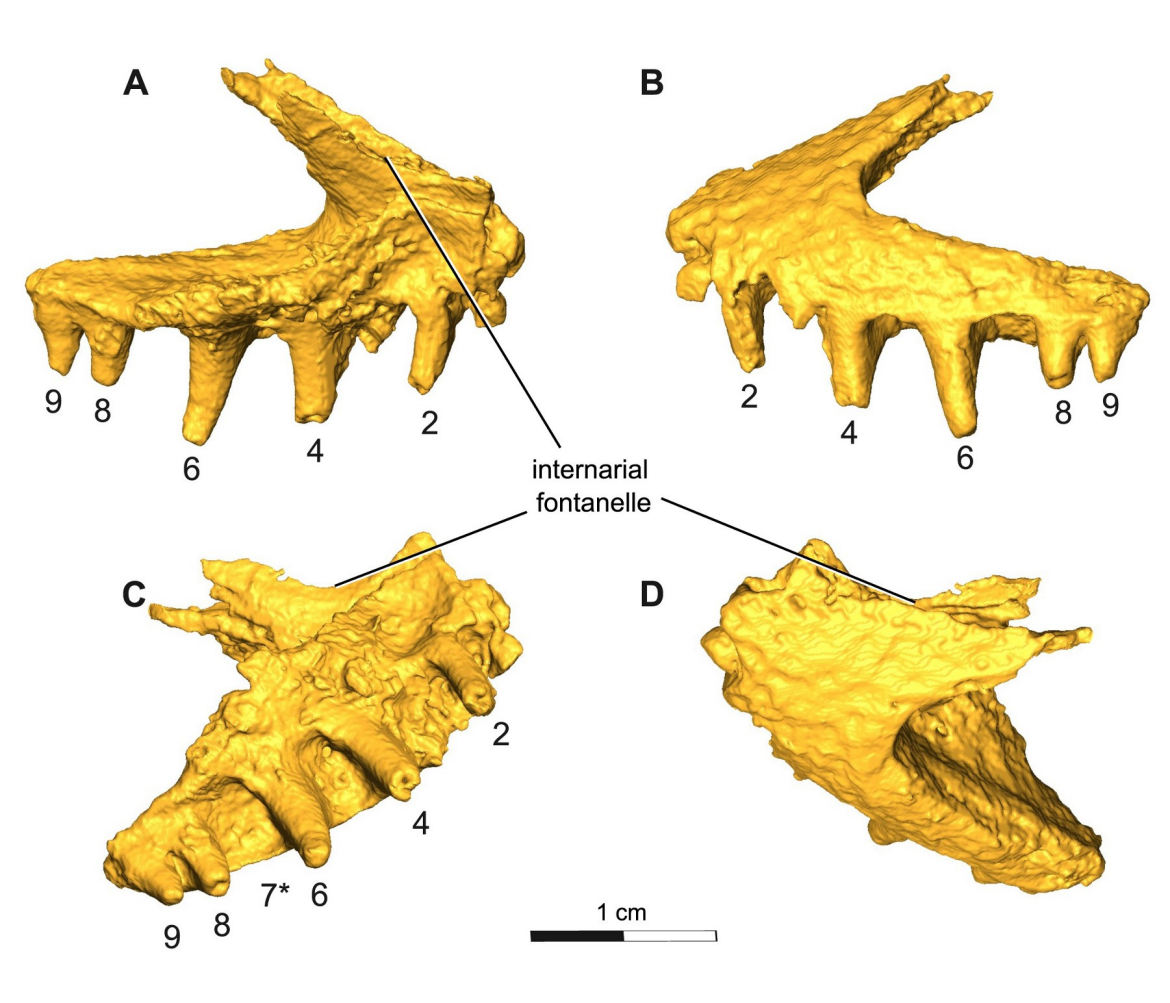
Segmentation rendering of *Acheloma cryptatheria* (ROMVP 88291) left premaxilla from CT image sequence. Shown in (A) medial view, (B) lateral view, (C) ventral view, and (D) dorsal view. Dentition numbered in sequential order, where position seven is labelled as “7*” for caniniform tooth position based on the large relative size of the tooth socket compared to the rest of the tooth row. Scale bar equal to 1 cm.

The premaxilla is a triangular element with a well-developed alary process extending posterodorsally towards the nasals anterolateral margin, similar to that of the juvenile trematopid, OMNH 79318, previously assigned to *Acheloma* [11]. The premaxilla is slightly disarticulated, but would contact the maxilla ventrolaterally and the vomer ventromedially, forming the anterior margin of the external naris dorsolaterally, as well as the anterior half of the intervomerine fossa ventrally. Although there is a missing right premaxillary element, a large embayment is still preserved along the dorsal midline between the premaxilla and nasal elements. This large embayment has a closer resemblance, size, and positioning to the internarial fenestra seen in *A*. *cumminsi* than to the condition in the holotype *A*. *dunni*, in which this opening is greatly reduced and excludes the nasal bone from it. A notable feature of this emargination is the presence of an internal shelf that forms a lateral wall of the narial fenestra. There are at least nine tooth positions in ROMVP 88291. They successively increase in size posteriorly until the largest tooth in position seven, and thereafter gradually decrease in size to the end of the row, indicating the presence of an enlarged “caniniform”, a condition typical of trematopids (Fig 4). The tooth count is similar to that seen in *A*. *cumminsi* [7] and is well below the thirteen tooth positions present in *A*. *dunni* [10]. The premaxillary dentition appears as sharply pointed, slightly recurved cones.

The maxilla is a long, slender dentigerous element that sutures to the premaxilla anteriorly, to the lacrimal dorsally, and to the palatine ventromedially, forming most of the ventral margin of the external naris. It also contacts both the lateral exposure of the palatine and ectopterygoid dorsally. In *Acheloma dunni* and in *A*. *cumminsi*, the maxilla forms a dorsal contact with the jugal and a posterior contact with the quadratojugal. This, however, cannot be clearly identified in ROMVP 88291, due to the fragmentation and displacement of the left quadratojugal and the posterior end of the right maxilla. The greatest dorsal expansion of the maxilla is also beneath the external narial opening and is where the largest maxillary teeth are situated. As the maxilla moves posteriorly, past the dorsal contact with the lacrimal, this expansion gently tapers to about half its original size, similar to the condition seen in *A*. *dunni*. The left maxilla is complete, displaying twenty-three tooth positions. The largest of the maxillary teeth are peg-like and monocuspid, whereas the smaller teeth are more similar in shape to the premaxillary dentition. Typical trematopid enlarged “caniniform” dentition is present on the left maxillary element in the fifth and sixth tooth positions, which is situated anterolaterally from the palatal fangs. *A*. *dunni* [10] shows caniniform teeth in the sixth and seventh tooth position, whereas *A*. *cumminsi* [7] appears to show them in the fifth and sixth position. The caniniform teeth in ROMVP 88291 are therefore consistent with *A*. *cumminsi* and are positioned farther anteriorly on the maxilla and anterior palatal fang when compared to the dentition found in *A*. *dunni*.

**Fig 5:**
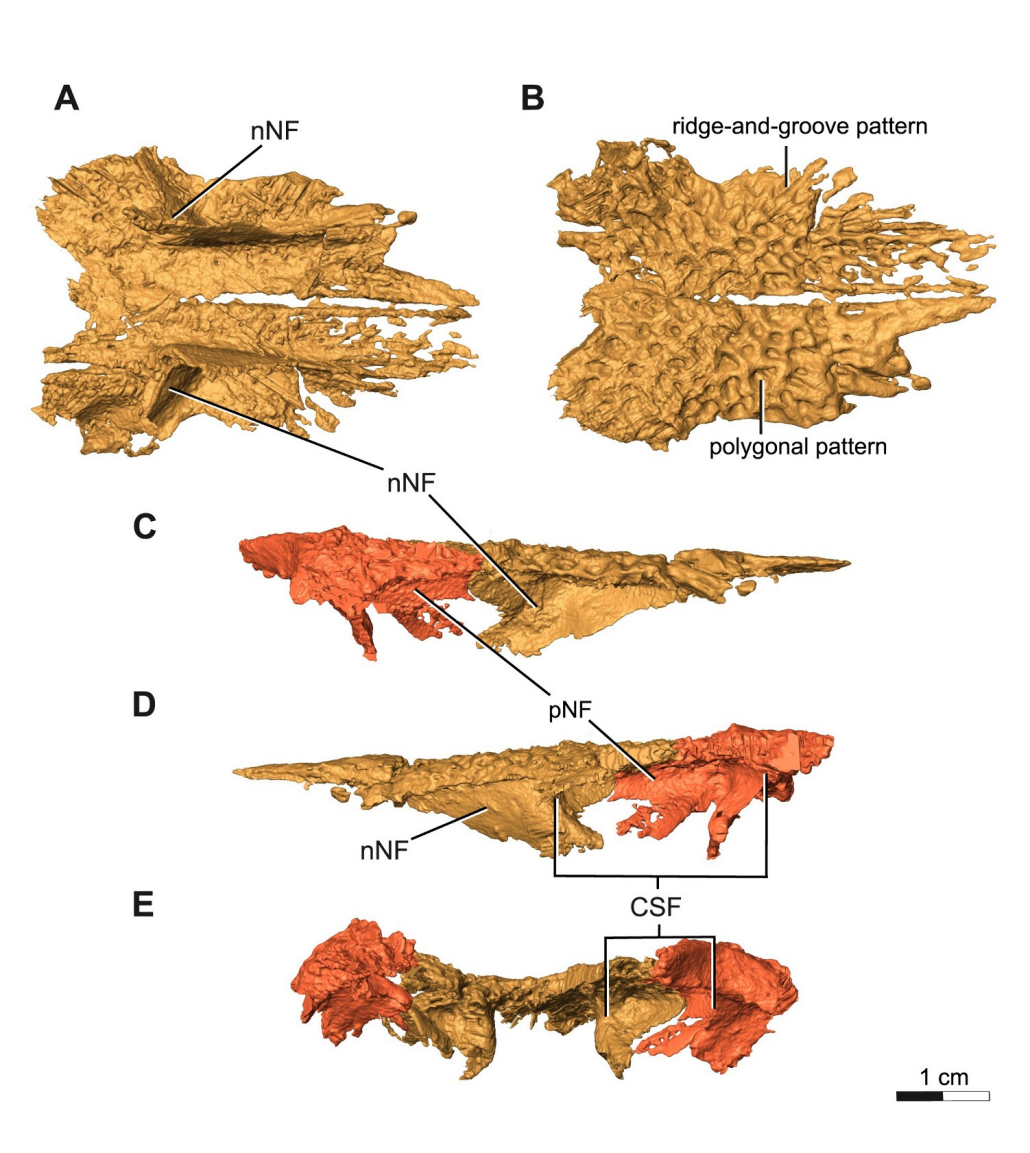
Segementation rendering of *Acheloma cryptatheria* (ROMVP 88291) right & left nasal from CT image sequence. Both nasal shown in (A) ventral view and (B) dorsal view. Prefrontals included in (C) right lateral view, (D) right medial view, and (E) occipital view for the purposes of highlighting the entire structure of the concave shelf formed with the nasals. Abbreviation: CSF, concave shelf/secondary chamber; nNF, nasal contribution of narial flange; pNF, prefrontal contribution of narial flange; r&g, ridge-and groove. Scale bar equal to 1 cm.

The nasal is a rectangular, flat elongate element that contacts the premaxilla anteriorly, prefrontal posterolaterally, and frontal posteriorly. It frames most of the dorsal roof of the elongated external nares, creating a bell-shaped dorsolateral expansion that combined with the dorsal expansion of the maxilla produces a “key-hole” morphology of the elongated external nares. This is one of many defining features of the clade Trematopidae [3,4,26–28]. A ventral flange on the nasal contributes the most to the narial flange and appears to contact the median vomerine septum of the vomer. However, it is difficult to be confident about this contact because it may be a result of flattening of the skull roof. Contact between the median vomerine septum and the skull roof has only been confirmed for *A*. *cumminsi*, but has not been described in the case of *A*. *dunni* [10]. In addition to its anterior contribution to the narial flange, the nasal also forms the anteromedial half of a concave shelf located in between the narial flange structure and the orbital wall, clearly visible in both medial and occipital view (Fig 5D and 5E).

**Fig 6:**
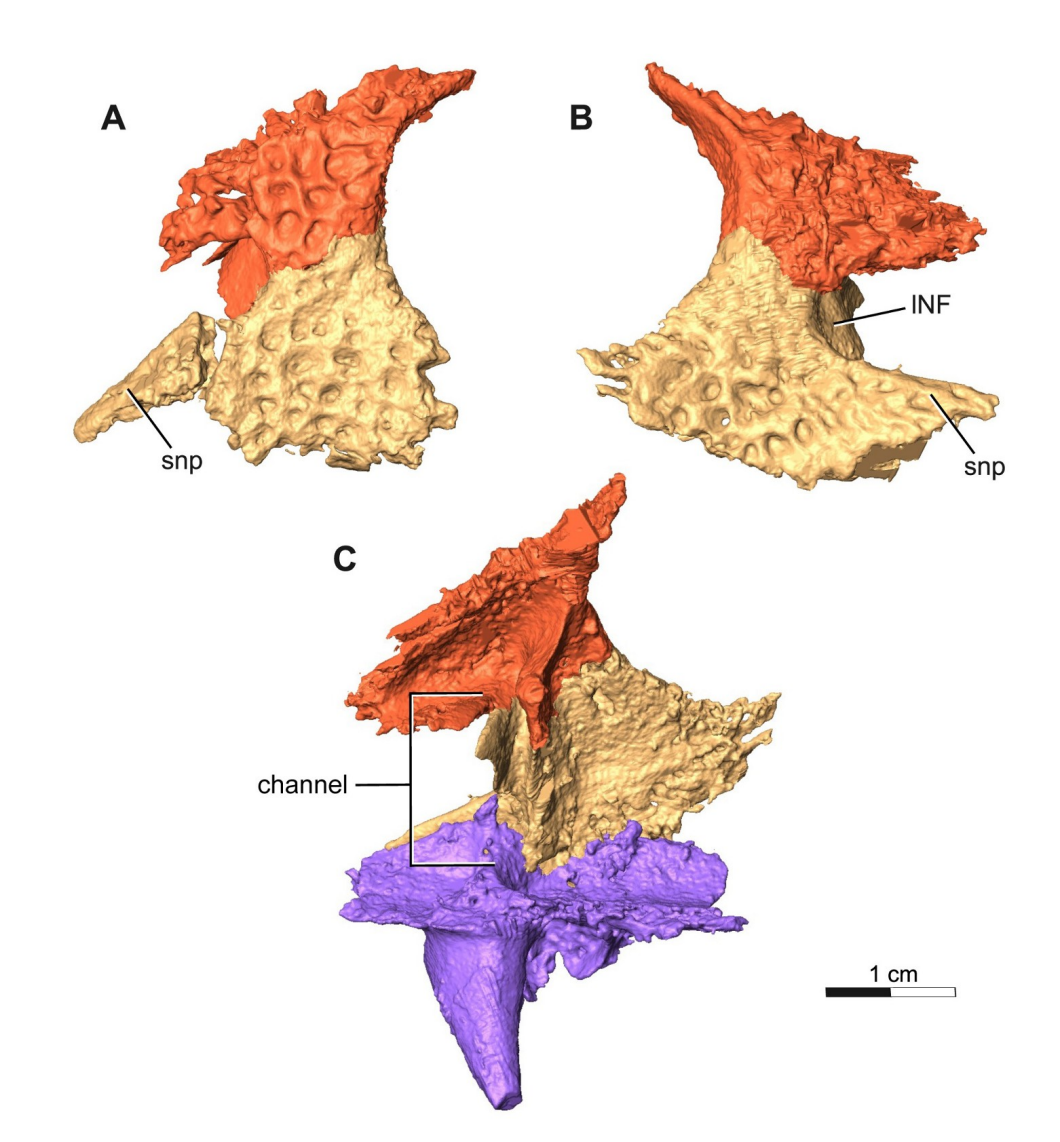
Segmentation rendering of *Acheloma cryptatheria* (ROMVP 88291) left & right lacrimal from CT image sequence. This is shown in association with the prefrontal in (A) left lateral view and (B) right lateral view, as well with the palatine in (C) right medial view for the purpose of showing the extent of the subnarial process and the entire channel. Abbreviation: lNF, lacrimal contribution of narial flange; snp, subnarial process of lacrimal. Scale bar equal to 1 cm.

The prefrontal is a triangular, flat element that contacts the nasal anteromedially, the frontal posteromedially, and lacrimal ventrolaterally. Anteriorly, this element contributes to the narial flange, as well as the posterodorsal half of the elongate external nares. Posteriorly, the prefrontal forms the anterodorsal margin of the orbit and the dorsal half of the antorbital bar. In addition to this, the prefrontal also contributes to the posterolateral half of the concave shelf found directly posterior to the narial flange (Fig 5D and 5E). The two walls of this concave shelf clearly protrude medially to form this deep chamber, but also deflects ventrally to form the walls of what appears to be a channel which connects to this secondary chamber (Fig 6C).

The lacrimal is a slender, flat, triangular element that contacts the prefrontal dorsally and the maxilla anteroventrally on the left side, but appears to contact the maxilla ventrally on the more complete right half. The lacrimal also contacts the palatine posteroventrally, and jugal posterodorsally. It contributes the least to the narial flange and forms the posteroventral half of the elongate external nares. It also contributes to the anteroventral margin of the orbit and the ventral half of the antorbital bar. Although disarticulated from the rest of the bone, the anterior tip of the lacrimal, also referred to as the subnarial process, appears to be an elongate extension that reaches nearly as far as half of the length of the elongate external nares. This relatively elongated subnarial process (as seen in Fig 6) resembles that of *Acheloma cumminsi* and other trematopids (refer to Discussion), in contrast to the short subnarial process seen in *A*. *dunni*. Similar to the prefrontal, two flanges protrude medially to form the walls of a channel that connect the dorsal floor of the palatine with the secondary chamber formed by the prefrontal and nasal (Fig 6C). As a result of its close proximity to the internal naris floored by such palatal features [16], it may be suggested that this channel and secondary chamber are possibly related to nasopharyngeal breathing or other nasal system functions.

The frontal is a flat, rectangular element that contacts the nasal anteriorly, the prefrontal anterolaterally, parietal posteromedially, and postfrontal posterolaterally. The sutural pattern formed between the frontal and the surrounding elements more closely resembles that of *Acheloma dunni*, as opposed to *A*. *cumminsi*, in that the prefrontal does not intrude into it but still extends posteriorly across its lateral margin, nearly excluding it from the orbital margin. The parietal also tapers into the frontal along the midline of the skull, similar to the suture pattern in *A*. *dunni* but not *A*. *cumminsi*. It also contributes slightly to the dorsal margin of the orbit.

The parietal is a trapezoidal element that contacts the frontal anteromedially, postfrontal anterolaterally, supratemporal laterally, and postparietal posteriorly. The sutural pattern between the parietals and the surrounding elements closely resembles that of *A*. *dunni*. The pair of parietals frame the pineal foramen directly along their medial suture. However, the relative size of this foramen is much larger than that of *A*. *dunni* and resembles more closely that of *A*. *cumminsi*.

The postfrontal is a roughly crescentic, quadrangular element that contacts the frontal medially, the parietal posteromedially, the supratemporal posteriorly, and the postorbital laterally. This element also contributes to the posterodorsal margin of the orbits and has sutural patterns that closely resemble both *Acheloma dunni* and *A*. *cumminsi*.

The postorbital is a crescentic element that contacts the postfrontal dorsally, the supratemporal and squamosal posteriorly, and the jugal ventrally. It makes a large contribution to the posterior margin of the orbits and has sutural patterns that closely resemble both *Acheloma dunni* and *A*. *cumminsi*. Strangely, the left postorbital of ROMVP 88291 is much more elongate and slender than the right postorbital.

The squamosal is a large, complex, crescent-shaped element that contacts the postorbital anteriorly, jugal anteroventrally, quadratojugal posteroventrally, and supratemporal dorsally. Similar to the condition in both *Acheloma* species, this element appears to extend considerably posterodorsally towards a contact with the tabular and with the quadrate posteriorly. The squamosal also contributes greatly to the formation of the otic notch, which is framed by the non-ornamented, medially inflected dorsal and ventral flanks of the supratympanic flange. This ventral flank, as well as the ventral margin of the otic notch, is almost exclusively formed by the squamosal. This ventral flank extends posteriorly, parallel with the dorsal flank, and then gently descends and tapers off at an overlapping contact with the quadratojugal. This sloping of the squamosal prevents the structure from developing the distinct “semilunar curvature” observed in many dissorophids and trematopids, with the exception of both *Acheloma dunni* and *A*. *cumminsi* [7,8,10,13,29]. In addition, the squamosal also contributes a small portion to the dorsal flank of the supratympanic flange, which includes part of the unsculptured dorsal margin of the otic notch.

The quadratojugal is a large “tear-drop” shaped element that contacts the squamosal dorsally, jugal anterodorsally, lateral exposure of the ectopterygoid anteriorly, and maxilla anteroventrally. It also would contact the quadrate posterodorsally and the articular posteriorly, but these elements are either missing or extremely fragmented in ROMVP 88291. The most notable feature of this element is the direct contact it makes with the LEE (lateral exposure of the ectopterygoid). This contrasts the condition seen in *Acheloma cumminsi* (refer to Gee et al. [11], Fig 5), as well as *A*. *dunni*, in which the two elements are separated by the jugal. In addition, the quadratojugal frames much of the lateral margin of the adductor fossa.

The supratemporal is a box-shaped element that contacts the postfrontal and postorbital anteriorly, squamosal ventrally, tabular posterolaterally, postparietal posteromedially, and parietal medially. The supratemporal contributes to the dorsal flank of the supratympanic flange within the unsculptured dorsal margin of the otic notch. It does not, however, extend posteroventrally in the same as seen in the squamosal or tabular.

The tabular is an elongate, crescent-shaped element that contacts the supratemporal anteriorly and the postparietal medially. This element also contributes to dorsal flank of the supratympanic flange and therefore the posterodorsal end of the otic notch. The posteromedial end of the tabular contributes to the occipital flange, best seen dorsal to the foramen magnum in occipital view. Unlike the supratemporal, the tabular is the portion of the dorsal margin of the skull and otic notch that extends posteroventrally toward the quadrate. In both *Acheloma dunni* and *A*. *cumminsi* holotypes, the tabular is shown to contact the quadrate and the posterior end of the quadratojugal. Judging by the large size of the tabular and its relative closeness to the quadratojugal and quadrate, it is likely that they would have also contacted and closed the posteroventral end of the otic notch.

The quadrate is extremely fragmented and contacts the quadratojugal laterally and is located at the posterolateral most portion of the skull. In both species of *Acheloma*, the quadrate would contact the quadrate ramus of the pterygoid medially, the posterior end of the tabular horn dorsally, and the squamosal anteriorly. Together with the quadratojugal, the slightly convex ventral surfaces would have accompanied the slightly convex dorsal surface of the articular to form the jaw articulation. In addition, the anterior surface of the quadrate retains a similar concavity as the medial surface of the quadratojugal, suggesting that it formed the posteromedial margin of the adductor fossa.

The postparietal is a flat rectangular shaped element that contacts the parietal anteriorly, supratemporal anterolaterally, and tabular posterolaterally. The posteroventral projection of this element contributes to the occipital flange which frames the dorsal margin of the foramen magnum. This occipital flange is where the postparietal contacts the underlying exoccipitals, as seen in all trematopids.

### Palate

**Fig 7:**
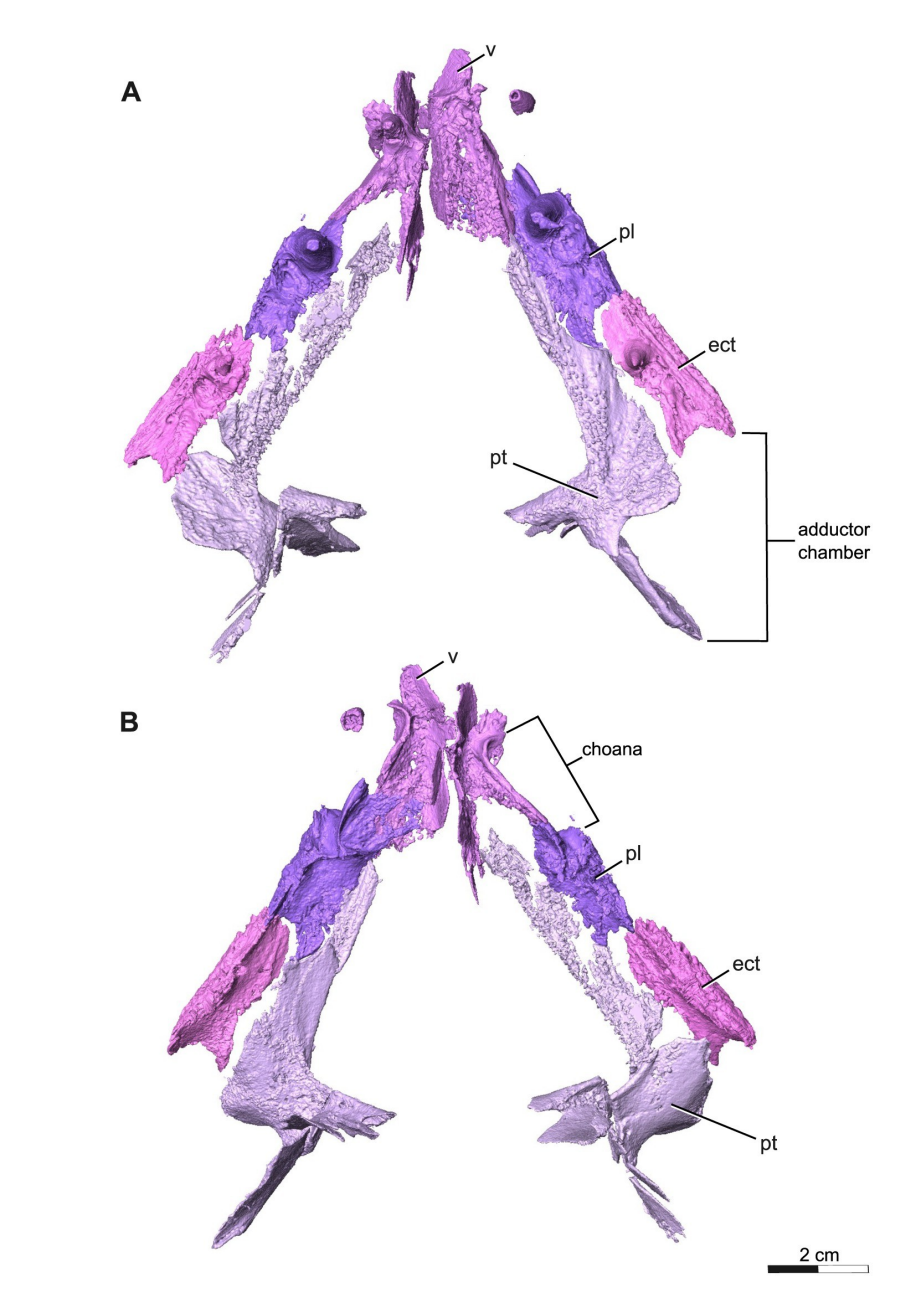
Segmentation rendering of *Acheloma cryptatheria* (ROMVP 88291) palate from CT image sequence. These are shown in (A) ventral view and (B) dorsal view. Abbreviation: ect, ectopterygoid; pl, palatine; pt, pterygoid; v, vomer. Scale bar equal to 2 cm.

**Fig 8:**
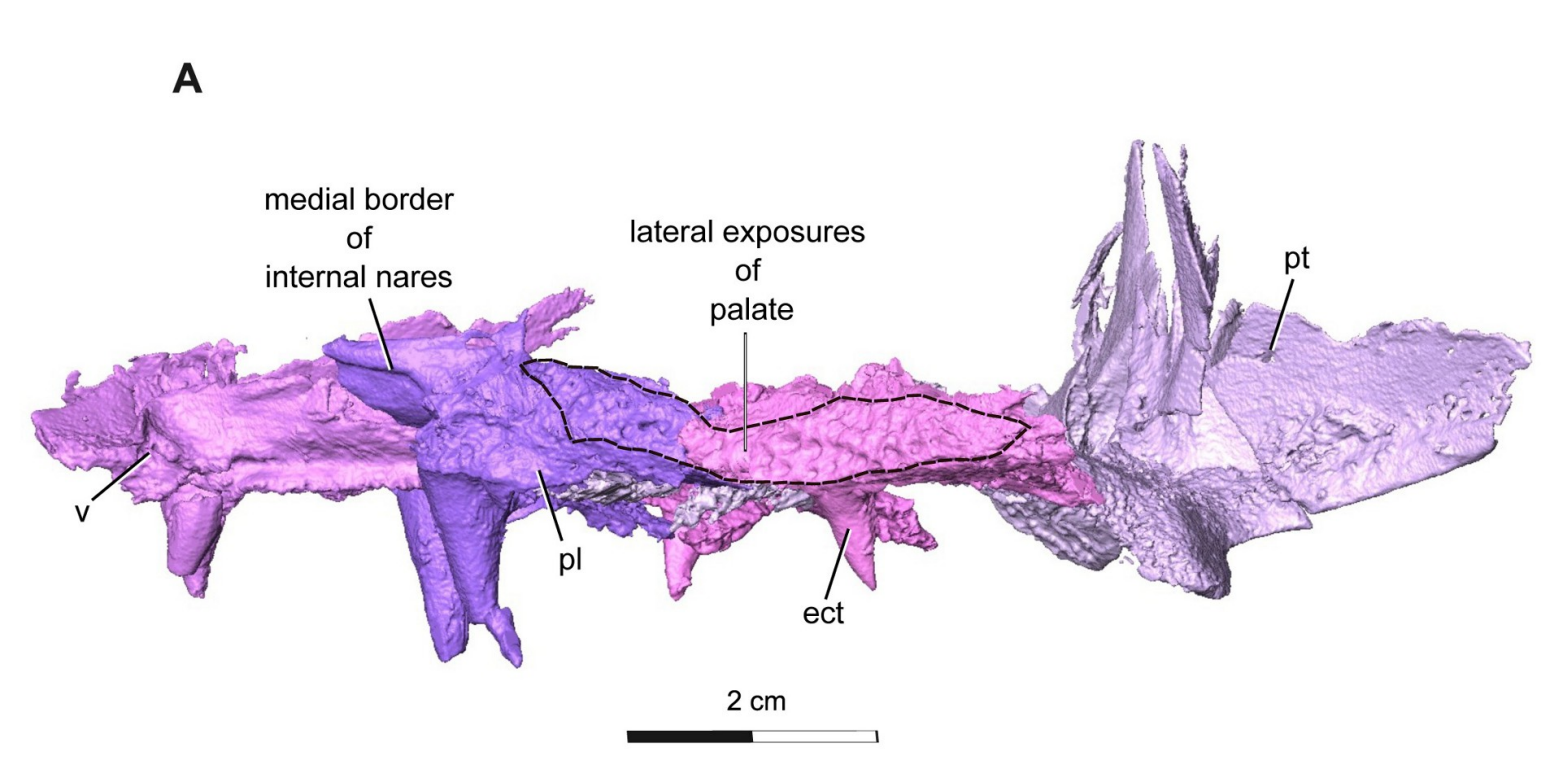
Segmentation rendering of *Acheloma cryptatheria* (ROMVP 88291) lateral palate view from CT image sequence. Shown in (A) left lateral view. Dark dashed lines highlighting the full extent of the lateral exposures relative to their respective elements. Abbreviation: ect, ectopterygoid; pl, palatine; pt, pterygoid; v, vomer. Scale bar equal to 2 cm.

The palate is completely represented and remains articulated in ROMVP 88291 with exception of the parasphenoid. It is covered by several denticle fields and has a total of twelve tusks/fangs (as seen in Figs 7 and 8).

**Fig 9:**
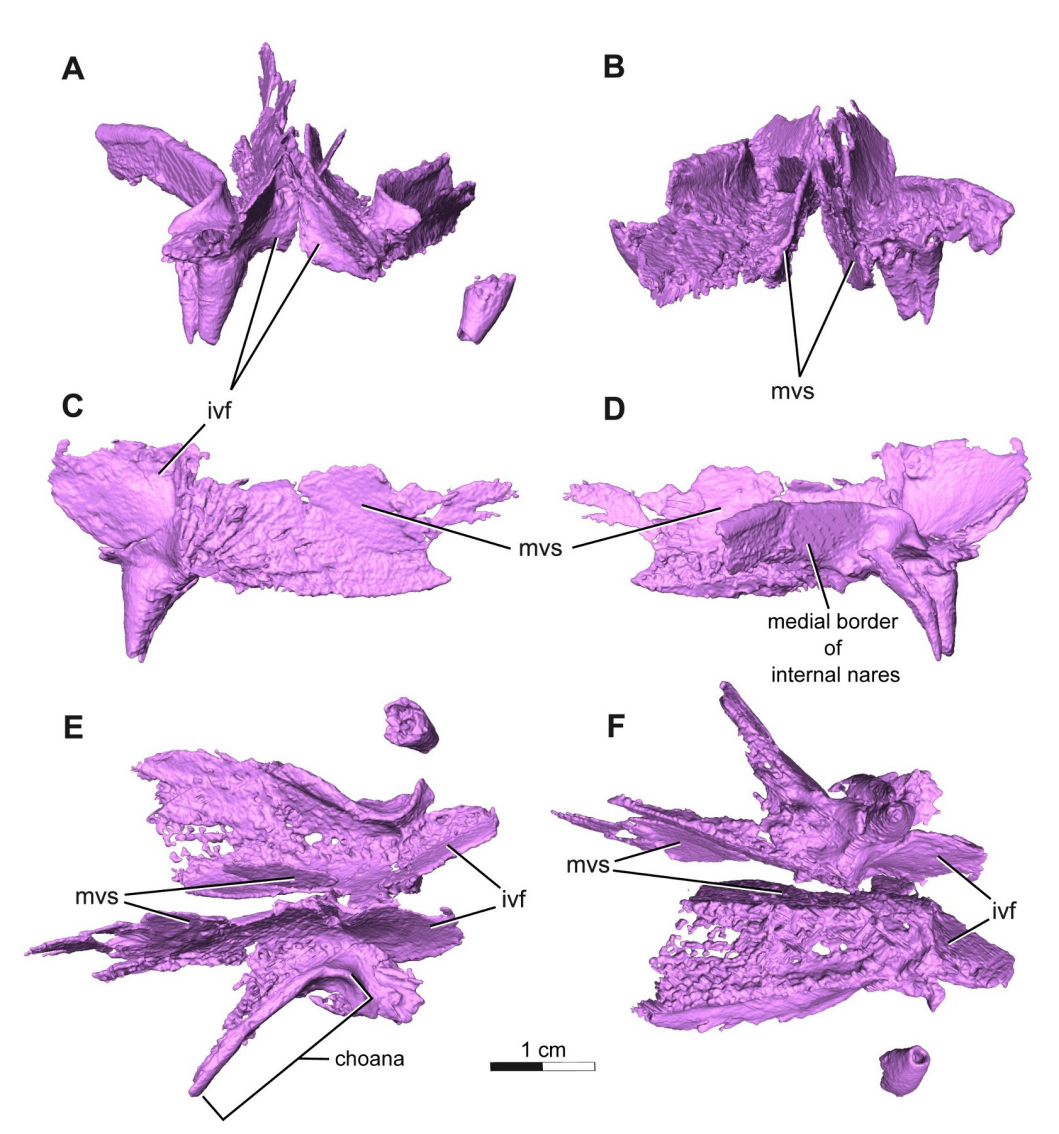
Segmentation rendering of *Acheloma cryptatheria* (ROMVP 88291) left & right vomer from CT image sequence. Shown in (A) anterior view, (B) posterior view, (C) right medial view, (D) right lateral view, (E) dorsal view, and (F) ventral view. Abbreviation: ivf, intervomerine fossa; mvs, medial vomerine septum. Scale bar equal to 1 cm.

The vomer is a slender rectangular element that contacts the premaxilla anterolaterally, palatine posterolaterally, pterygoid posteromedially. Ventrally, the anteromedial margins of the vomers deflect dorsally to contribute to the posterior half of the intervomerine fossa, the lateral margin contributes to the anteromedial half of the choana, and the posterior end to the anterior margin of the interpterygoid vacuity. A raised dentigerous crest along the medial border of the choana is present on the left vomer and is also exhibited in some primitive temnospondyls [24]. It is unclear if the vomer of ROMVP 88291 displays a y-shaped choana, since this is the typical condition for *Acheloma* and distinguishes them from other trematopids possessing an oval-shaped choana [11,21]. A single pair of slightly recurved, monocuspid, and robust fangs are present on the anterolateral process of the right vomer and two adjacent replacement pits are seen on the left vomer where the corresponding pair of vomerine tusks would be (Fig 9F). Smaller pairs of fangs are typically found posterior to these larger pairs and would be longitudinally oriented along the raised crest of the medial border of the choana; however these do not appear to have been preserved on either vomer in ROMVP 88291. It is likely that these smaller fangs would have been present based on the many similarities ROMVP 88291 has shown with the other *Acheloma* species, which do possess them. A dentigerous ridge located posterior to the large pair of vomerine fangs extends towards the posterolateral edge of the vomer where it meets the pterygoid and palatine bones. This placement and anteroposterior extension of the dentigerous ridge on the vomer is considered to be an autapomorphy of *Acheloma* sensu Gee 2020 [10,11,21]. On the dorsal surface, the medial process running along the midline deflects and extends posterodorsally to form the median vomerine septum (Fig 9A and 9B), where it meets the ventral surface and ventral flange of the nasal dorsally, and would contact the cultriform process of the parasphenoid posteriorly if it were preserved. A contact between the skull roof and the median vomerine septum may have functioned as a barrier separating the two nasal chambers. In ROMVP 88291 this septum contacts the skull roof, but this may be due to the flattening of the skull that forced this contact, meaning that the original closeness between these two nasal chambers is uncertain. The lateral margin of the vomer, which also frames the choana, deflects dorsally in a similar manner to the medial process. It may be suggested that the crevice-like pathway that are formed by these dorsally deflected processes may have carried the unusually elongate olfactory tract, if the olfactory bulbs were located far anteriorly.

**Fig 10:**
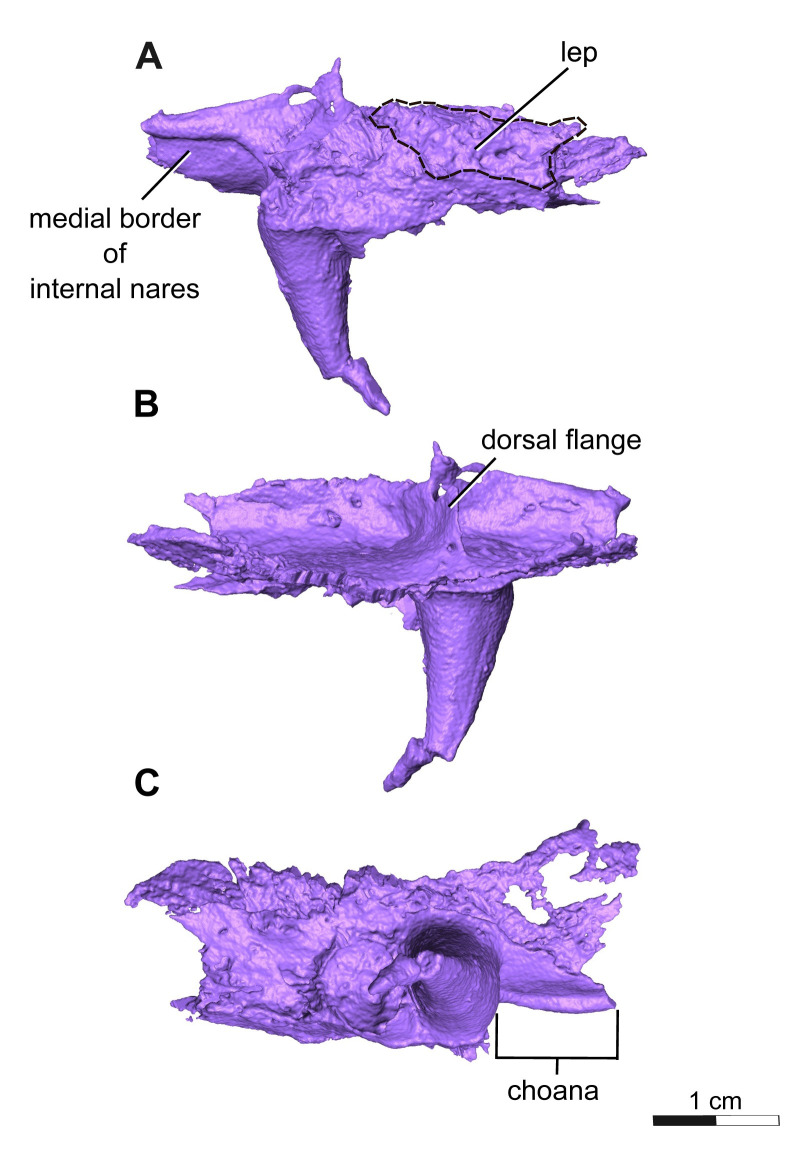
Segmentation rendering of *Acheloma cryptatheria* (ROMVP 88291) left palatine from CT image sequence. Shown in (A) left lateral view, (B) left medial view, and (D) left ventral view. White dashed lines highlight the exposed area of the palatine. Abbreviation: lep, lateral exposure of the palatine. Scale bar equal to 1 cm.

The palatine is a rectangular element that contacts the vomer anteromedially, maxilla ventrolaterally, ectopterygoid posteriorly, pterygoid medially, and lacrimal dorsolaterally. The anterior end of this element also frames the posterior margin of the choana along with the vomer and maxilla, but to a lesser extent than what is seen in *Acheloma dunni*. Two large fangs would be present on the ventral surface of the palatine directly medial to the sixth and tenth maxillary teeth, although only one large fang is preserved on each followed by a slightly smaller fang for which only the tooth socket remains. The fang on each palatine is robust, monocuspid, and slightly recurved, as described above, but are about twice the diameter and length of the vomerine and ectopterygoid fangs. Oddly, the ventral surface of the palatine appears to be devoid of the smaller dentition found on the vomer and pterygoid bones (Fig 10C). The dorsal surface of the palatine is also quite peculiar in that a dorsal flange extends towards the medial flanges of the lacrimal, thereby completing the channel structure that it forms along with the prefrontal. Of particular note is the substantial lateral exposure of the palatine (LEP), which appears elongate, oval-shaped, and exhibits the same cranial ornamentation as the other skull roof elements around the orbit (Fig 10A). The lateral exposure lies dorsal to the maxilla and ventral to the lacrimal and jugal, and anterior to the lateral exposure of the ectopterygoid (LEE).

The ectopterygoid is another rectangular element of the palate that contacts the palatine anteriorly, maxilla ventrolaterally, pterygoid medially, and jugal dorsolaterally. The posterior rim of the ectopterygoid also frames the anterior edge of the adductor fossa. The ectopterygoid resembles the palatine, in possessing two large palatal fangs medial to the seventeenth and twentieth maxillary teeth. Only the first larger fang is preserved, followed posteriorly by a slightly smaller fang represented by an empty socket. These fangs are roughly half the size of the palatine fangs, but still appear more exaggerated than the marginal maxillary teeth. This is similar to the condition seen in *Acheloma dunni*, where a large size discrepancy exists between the ectopterygoid fangs and the marginal teeth, as opposed to the condition in *A*. *cumminsi* in which they are not significantly larger [7,10]. The lateral exposure of the ectopterygoid (LEE) is also rather noteworthy, in being considerably larger than that of the palatine. It is similar in shape to that of the palatine in being elongate, oval-shaped, and exhibiting the same cranial ornamentation as the other skull roof elements of the orbit. The lateral exposure is dorsal to the maxilla, ventral to the lacrimal and jugal, and directly posterior to the lateral exposure of the palatine.

The pterygoid is a large, irregularly shaped element with the entire ventral surface sporting dense dental fields, with the exception of the most posterior extension of the quadrate ramus. The palatine ramus extends anteriorly towards the vomer, gradually tapering mediolaterally past the lateral contact with the palatine and ectopterygoid, while also framing the lateral margin of the interpterygoid vacuity medially. This differs from that of *Acheloma dunni* where the process remains relatively the same size throughout its entire anteroposterior length, and rather more closely resembles the palatine ramus seen in smaller trematopids like *Phonerpeton pricei* [8] and a juvenile *Acheloma* specimen [11], despite being significantly larger than in the latter two. Posterior to the palatine ramus, is the transverse flange of the pterygoid, which curves posteroventrally to frame much of the anterior margin of the adductor fossa alongside the posterior most extent of the ectopterygoid. The basipterygoid ramus extends medially and would meet the parasphenoid at the basicranial articulation, which is not preserved. This process would also contribute to the straight posterior margin of the interpterygoid vacuity, suggesting that the shape of the vacuity may have been relatively trapezoidal, similar to the vacuity seen in *A*. *dunni*, as opposed to being more oval [8,11]. Although the parasphenoid is not preserved, the lateral outline of the interpterygoid space would not be affected by this dividing component of the vacuities. The quadrate ramus extends posterolaterally to suture to the quadrate, while also framing much of the medial margin of the adductor fossa. In addition, there is a flange that originates from the position of the basipterygoid ramus and extends posterodorsally onto the quadrate ramus, in close proximity to the medial interior of the squamosal.

### Mandible

**Fig 11:**
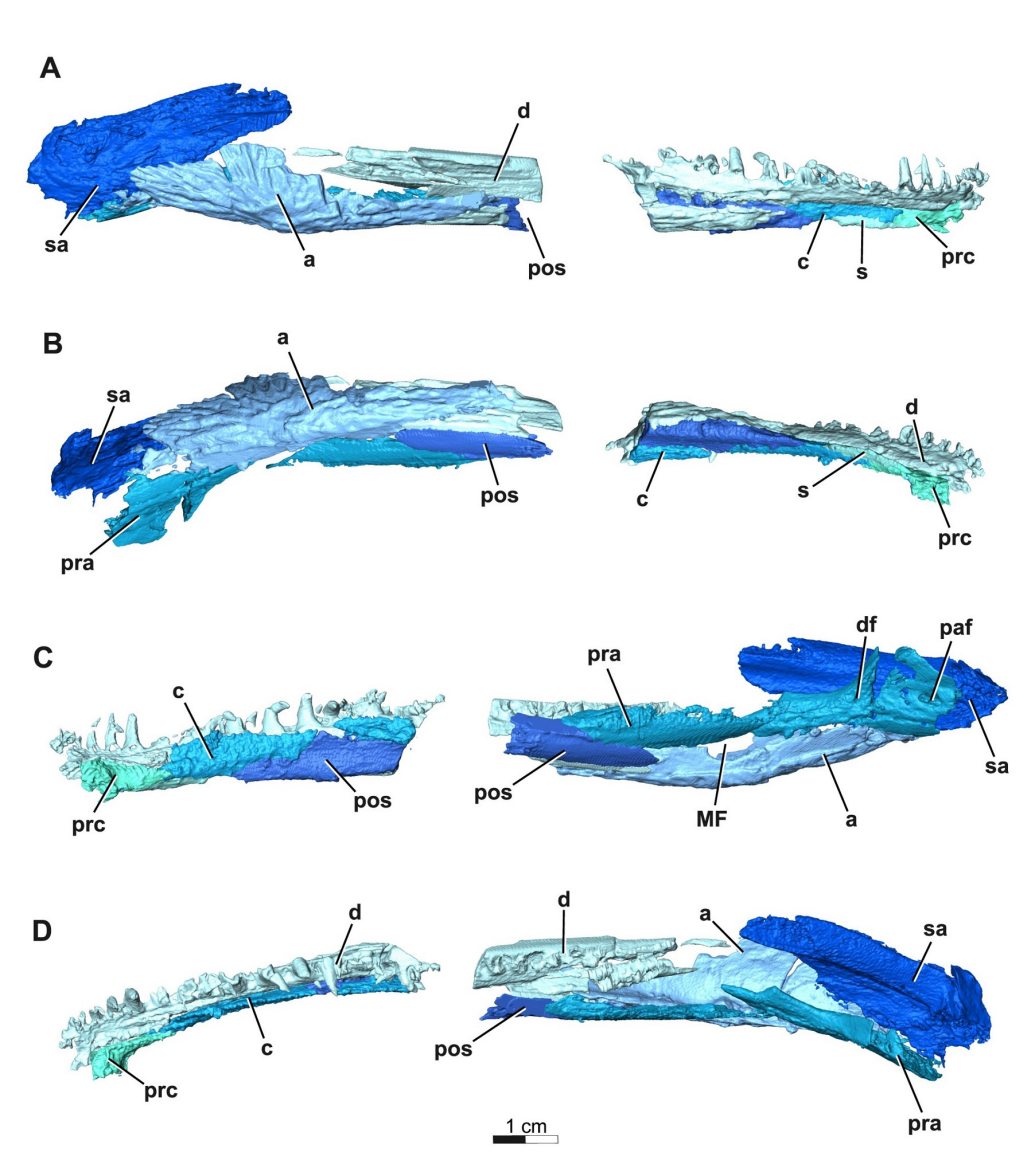
Segmentation rendering of *Acheloma cryptatheria* (ROMVP 88291) right mandible from CT image sequence. These are shown in (A) lateral view, (B) ventral view, (C) medial view, and (D) occlusal view. Abbreviation: a, angular; c, coronoid; d, dentary; df, dental foramen; MF, Meckelian foramen; paf, pararticular foramen; pos, postsplenial; pra, prearticular; prc, precoronoid; s, splenial; sa, surangular. Scale bar equal to 1 cm.

Only a partial, rather fragmentary right mandible is preserved in ROMVP 88291 (as seen in Fig 11), thereby limiting the amount of informative anatomical data.

A fragmented right dentary is largely represented. It contacts the precoronoid anteromedially, coronoid dorsomedially, splenial ventromedially, postsplenial ventrally, surangular posterodorsally, and angular posteroventrally. There are at least 23 tooth positions preserved in the right dentary, but there certainly would be more. Only one empty enlarged symphysial tooth socket is preserved in the most anterior tip of the dentary posteromedial to the first tooth in the marginal tooth row of the dentary. The remainder of teeth in the marginal tooth row are conical, mediolaterally broad, recurved and posteromedially tilted (with a more dramatic medial tilt) and appear to increase in tooth size posteriorly from the first to the twelfth tooth position, followed by a gradual posterior decrease in the subsequent teeth.

The precoronoid is a rectangular medial mandibular element that contacts the symphysial region of the dentary anteriorly, coronoids posterodorsally, and splenial posteroventrally. Most of the element is smooth, with the exception of the dorsal surface which preserves a denticulate texture.

The coronoid is a flat, rectangular element comprising most of the dorsomedial region of the mandible that contacts the dentary laterally, precoronoid anteriorly, splenial anteroventrally, and postsplenial posteroventrally. Typically, the coronoid extends posteriorly to contact both the surangular and prearticular, but most of the posterior extent of the coronoid is not preserved in ROMVP 88291. In addition to these contacts, the coronoids are also distinct from other mandibular elements for being entirely enveloped in a denticulate texture.

The splenial is a flat, rectangular element that contacts the dentary laterally, precoronoid anterodorsally, coronoid posterodorsally, and postsplenial posteriorly. Interestingly, the size of the splenial relative to the other elements is much smaller than that of other trematopids like *Acheloma dunni* and *A*. *cumminsi*, but this may reflect poor preservation.

The postsplenial is similar in shape to the splenial, however it is at least triple the size and contacts the dentary anterolaterally, coronoid dorsally, splenial anteriorly, angular posterolaterally, and prearticular posteriorly.

The prearticular is a long, thin element that contacts the postsplenial anteroventrally, angular ventrolaterally, surangular posterolaterally, and articular posteroventrally. It would also contact the coronoid anterodorsally to frame the adductor chamber, however the posterior extent of the coronoid is missing in this specimen. The prearticular is distinct from other mandibular elements in possessing a number of foramina along its ventral and posterior surfaces. One of these is the pararticular foramen (as seen in *Phonerpeton pricei* [8] and a juvenile *Acheloma* specimen [11]), located near the prearticular-articular contact on the posteromedial surface. Anterior to this foramen is the dental foramen, also noted by Dilkes [8] and Gee et al. [11]. Just posterior to the postsplenial-angular contact is the third foramen of the prearticular, the Meckelian foramen, and is ventrally framed by the angular and the prearticular dorsally.

The angular is a long, convex element that contacts the dentary anterolaterally, postsplenial anteromedially, prearticular posteromedially, and surangular posterodorsally. The lateral surface of this element also retains some of the same cranial ornamentation observed in the quadratojugal, but these longitudinal pits are tilted more posterodorsally towards the articular bone.

The surangular is a large, slender lateral mandibular element largely hidden medially by the quadratojugal and mostly contacts the angular ventrolaterally. It is not seen in ROMVP 88291, but it also contacts the coronoid anteriorly and the articular posteriorly. The surangular is also differentiated from other mandibular elements for having a forked anterodorsal surface, which would have mounted the posterior process of the dentary.

## Discussion

### A new *Acheloma* at Richards Spur

The description of ROMVP 88291 clearly indicates that this trematopid specimen exhibits many morphologies typical of *Acheloma* including the presence of a dentigerous crest on the vomer extending anteroposteriorly, a greater lateral exposure of the ectopterygoid (LEE) than that of the palatine (LEP) excluded from the orbit by a lacrimal-jugal contact, an otic notch which is dorsoventrally constricted, and a naris substantially longer than the orbit. ROMVP 88291 satisfies and maintains characters 1–3 of the Gee [21] revised diagnoses for *Acheloma*, but cannot confirm the fourth character related to an anteromedially expanded y-shaped choana due to poor preservation.

Interestingly, ROMVP 88291 appears more similar to *Acheloma cumminsi* but retains some features only found in *Acheloma dunni*. Given that the holotype of *A*. *dunni* and *A*. *cumminsi* (OMNH 73281 and AMNH 4205) are the largest known trematopid specimens and are nearly the same size as ROMVP 88291, they likely reflect similarly ontogenetically mature individuals. As such, direct comparisons can be made with reasonable confidence (Table 1) and that any differences are not a reflection of ontogenetic variation. The skull of ROMVP 88291 is proportionately shorter and wider than that of both *A*. *dunni* and *A*. *cumminsi*, whose skulls are slightly longer than they are wide. The maximum skull width also occurs at the level of the pineal foramen in ROMVP 88291, whereas in *A*. *dunni* and *A*. *cumminsi* maximum skull width occurs posteriorly to the foramen. The so-called y-shaped contour and elongation of the choana is difficult to view in the preserved remnant of the vomer, which is why this cannot be confidently compared. However, a comparison of *Acheloma* with other trematopids, such as *Phonerpeton pricei* [8], reveals that this y-shaped contour is likely a result of enlarged vomerine fangs on the anterolateral process of the vomer penetrating the anterior margin of the choana and effecting its shape. Although not clearly preserved, it is likely that ROMVP 88291 also displayed this morphology due to the sheer size of the preserved vomerine fangs relative to the anterolateral process.

**Table 1:** Summary table of morphological differences & similarities between ROMVP 88291, *Acheloma dunni* (OMNH 73281), and *Acheloma cumminsi* (AMNH 4205).

Feature	ROMVP 88291	*A*. *dunni*	*A*.*cumminsi*
Total midline skull length	~13.5 cm	16.4 cm	15.5 cm
Maximum skull width	~14.6 cm	~15.5 cm	~13.6 cm
Skull length:width	~0.92x	~1.05x	~1.14x
Diameter of orbit	~2.7 cm	~3.5 cm	~3 cm
Orbit:length	~0.2x	~0.21x	~0.19x
Orbit:width	~0.19x	~0.23x	~0.22x
Length nasal:frontal	~1.2x	~ 1.5x	~1.3x
Snout tip	Rounded	Rounded	Rounded
Internarial fontanelle size	Large	Small	Large
Position of LEP	Between lacrimal and maxilla	Between lacrimal and maxilla	Between lacrimal and maxilla
Position of LEE	Between jugal and maxilla	Between jugal and maxilla	Between jugal and maxilla
Alary process of premaxilla	Nasal extends anteriorly to contact the alary process medially ONLY (similar to Gee et al. [11] *Acheloma*)	Nasal extends anteriorly to envelop alary process medially and laterally	Nasal extends anteriorly to envelop alary process medially and laterally
Premaxillary dentition	~ 9 tooth positions	~ 13 tooth positions	~ 9 tooth positions
Maxillary dentition	At least 23 tooth positions	At least 28 tooth positions	At least 23–26 tooth positions
Maxillary caniniform dentition	5th & 6th tooth positions	6th & 7th tooth positions	5th & 6th tooth positions
Contact b/w median vomerine septum and nasal roof	?	?	Yes
Subnarial process of lacrimal	Long	Short	Long
Frontal sutures with surrounding elements	Resembles the suture patterns of *A*. *dunni*		
Parietal sutures with surrounding elements	Resembles the suture patterns of *A*. *dunni*		
Semilunar curvature	Absent	Absent	Absent
Contact between LEE and quadratojugal	Yes	No	No
Fused LEE	Yes	No (2 pieces)	Yes
Choana	Unclear (likely y-shaped based on size of tusks)	Y-shaped	Y-shaped
Ectopterygoid fangs	Larger than marginal teeth	Larger than marginal teeth	About the same size of marginal teeth
Anterior process of pterygoid	Tapers as it extends anteriorly towards vomer contact	Maintains its size throughout entire extension	?
Interpterygoid vacuity shape	Trapezoidal-shaped	Trapezoidal-shaped	?
Snout cranial ornamentation	Rounded & elongate pitting	Only rounded pitting	Rounded & oval pitting

Of particular significance are the defining placements and configurations of the lateral exposures of the palatine (LEP) and ectopterygoid (LEE). Most olsoniformes with these lateral exposures typically separate the lacrimal and jugal elements, thereby contributing to part of the orbital margin [11]. However, partly due to the large height of the suborbital bar, the lateral exposures seen in *Acheloma dunni* and *A*. *cumminsi* are situated between the maxilla and jugal, thereby excluding these exposures from the orbital margin and allowing the lacrimal and jugal to contact one another [11]. The overall size and placement of the lateral exposures described for ROMVP 88291 are distinct from both species of *Acheloma*. However, only one LEE is present as in *A*. *cumminsi*, and in contrast to the condition seen in *A*. *dunni* where two distinct LEE elements are present.

The presence of lateral exposures of the palatine (LEP) and/or ectopterygoid (LEE) are apparently vital in supporting the mechanical stresses some dissorophids (e.g. *Cacops morrisi* [30,31]) and trematopids (e.g. *Fedexia striegali* [32], *Phonerpeton pricei* [8], *Acheloma* [7,10], etc.) experienced during feeding [33]. Although their occurrence appears to be variable amongst olsoniformes, *Acheloma* is distinct in the organization of these lateral exposures and the relative elongation of its LEE (ref. Table 2). The LEE in ROMVP 88291 was more massive and longer than the diameter of the orbit, making it absolutely and proportionately larger than the LEE of *A*. *dunni* and *A*. *cumminsi*. This expansion of the LEE excludes the jugal and lacrimal from the maxilla through a unique quadratojugal and LEE contact, thereby making this an autapomorphy for *Acheloma cryptatheria*. This is significant because the coupling of the lateral exposures and tall suborbital bar in *A*. *dunni* were suggested to greatly counteract the stresses forced on the skull and palate by enlarged ectopterygoid fangs during feeding [10]. ROMVP 88291 shares all of these features with *A*. *dunni*, but the occurrence of its proportionately larger LEE suggests that the structural integrity of the skull may have been comparable to that of a larger trematopid like *A*. *dunni*. If this is true, then it may suggest that *Acheloma cryptatheria* may have occupied a distinct niche from *Acheloma dunni* as top predators, and may have been able to handle larger and stronger prey. This is supported in part by the differences between the ornamentation in *A*. *dunni* and *A*. *cryptatheria*, especially in concert with the difference in size of the internarial fenestra.

**Table 2:** Summary table of differences related to the lateral exposure(s) and internarial fontanelle of all trematopid taxa.

Trematopid taxon	Presence of L.E.P.	Presence of L.E.E.	Position of L.E.E. and/or L.E.P.	Size of L.E.E. relative to orbit diameter	Presence of Internarial Fontanelle
ROMVP 88291	Yes	Yes	Excluded from the orbital margin and separates the lacrimal and jugal from maxilla	~1.2x larger than the size of the orbit. L.E.E. size: ~3.2–3.4cm Orbit diameter: ~2.7cm	Present
***Acheloma cumminsi***	Yes	Yes	Excluded from the orbital margin and partially separates the lacrimal and jugal from maxilla	~⅔ the size of the orbit. L.E.E. size: ~1.8cm Orbit diameter: ~3cm	Present
***Acheloma dunni***	Yes	Yes	Excluded from the orbital margin and partially separates the lacrimal and jugal from maxilla	~1x larger than the size of the orbit. L.E.E. size: ~1cm + ~2.5 cm = 3.5cm Orbit diameter: ~3.5cm	Present
***Phonerpeton pricei***	Yes	Yes	Ventral margin of orbit. Between lacrimal and jugal, excluding maxilla from orbit	N/A	Present
***Phonerpeton whitei***	N/A	N/A	N/A	N/A	N/A
***Rotaryus gothae***	No	No	N/A	N/A	Absent
***Fedexia striegeli***	Yes	N/A	Ventral margin of orbit. Between lacrimal and jugal, excluding maxilla from orbit	N/A	Absent
***Tambachia trogallas***	No	No	N/A	N/A	Absent
***Anconastes vesperus***	No	No	N/A	N/A	Absent
***Actiobates peabodyi***	Yes	No	Ventral margin of orbit. Between lacrimal and jugal, excluding maxilla from orbit	N/A	Present (according to Eaton [27])
***Ecolsonia cutlerensis***	Yes	No	Ventral margin of orbit. Between lacrimal and jugal, excluding maxilla from orbit	N/A	Absent
***Mordex calliprepes***	Yes	No	Ventral margin of orbit. Between lacrimal and jugal, excluding maxilla from orbit	N/A	Possibly, however cannot confirm (Milner [1])
***Mattauschia laticeps***	Yes	No	Ventral margin of orbit. Between lacrimal and jugal, excluding maxilla from orbit	N/A	Absent

*Phonerpeton whitei* is excluded from this table because of the lack of informative data and figures needed for its inclusion.

**Fig 12:**
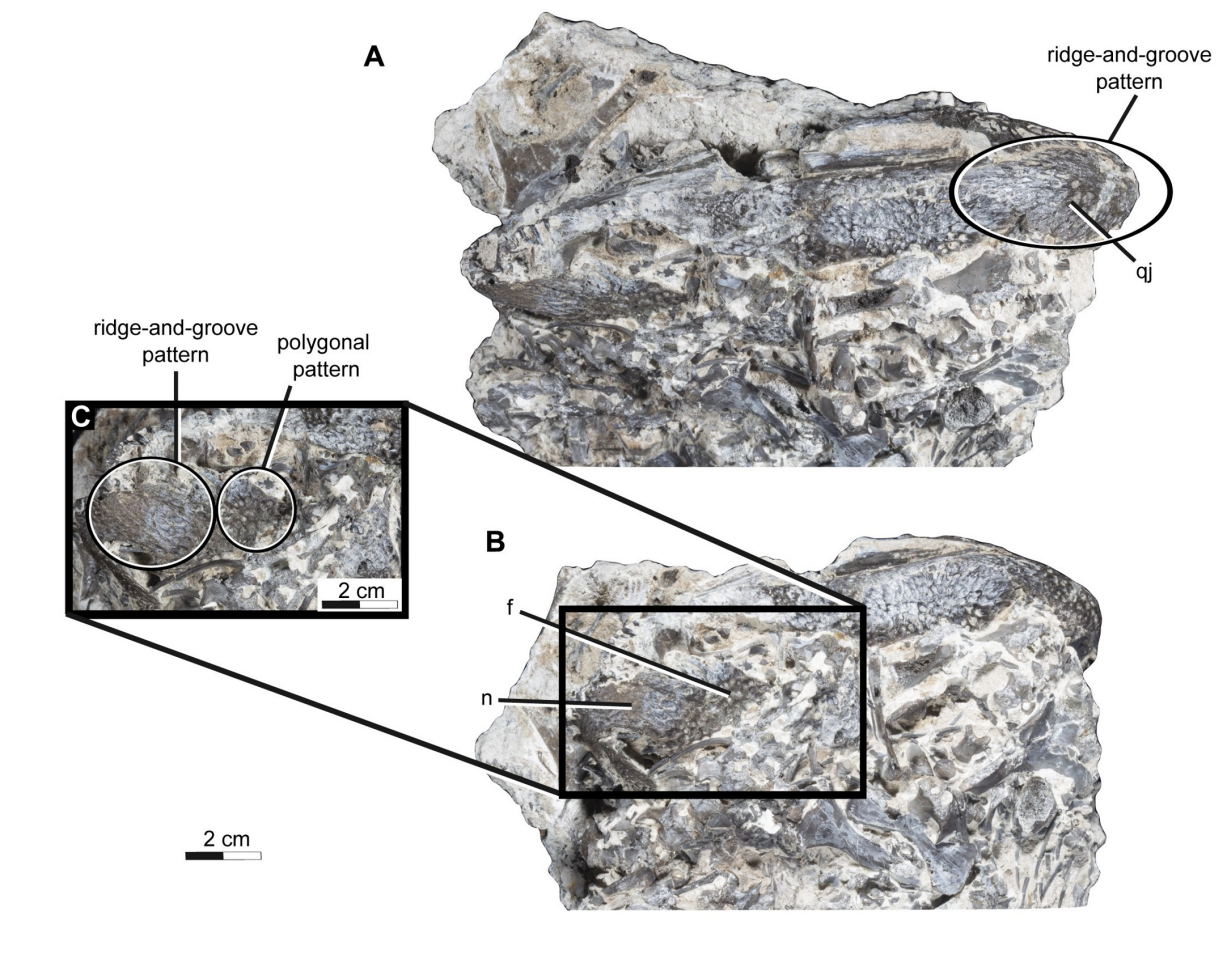
Photographs of *Acheloma cryptatheria* (ROMVP 88291) in dorsal & right lateral views. Shown in (A) right lateral view and (B) dorsal view. An additional magnified dorsal view of the snout (C) is shown to accompany the photographs outlining presence of ridge-and-groove morphology on exposed elements of the specimen. Abbreviation: f, frontal; n, nasal; qj, quadratojugal. Scale bar equal to 2 cm.

Many early amphibians including trematopids display a diverse array of cranial ornamentations. In trematopids, these ornamentations appear as a reticulate or polygonal/round pitting across the skull roof and are thought to serve some function in strengthening and dispersing stresses during biting [25]. It is apparent that there is some heterogeneity among the ornamentation of trematopids skulls, likely reflecting the distribution of various stresses produced by differing biting habits and/or prey choice. The polygonal/round cranial ornamentation extends across most of the skull roof except for the maxillae and premaxillae in both *Acheloma dunni* and *A*. *cumminsi*. While in *Acheloma cryptatheria*, the anterior most portion of the nasals and posterior most of edge of the quadratojugal and squamosal display an anteroposteriorly oriented parallel ridge-and-groove morphology (Fig 12), which is absent in *A*. *dunni* and weakly developed in *A*. *cumminsi* [7,25]. The polygonal/round versus the ridge-and-groove morphologies likely reflect omnidirectional and unidirectional distribution of stress respectively, which is also consistent with differences in the dentition [25]. It is likely that the former provides greater omnidirectional strength, appears to represent the primitive condition, and may also be more energetically costly to produce [25].

### Controversies surrounding *Acheloma*

Designation of ROMVP 88291 as a new species, *Acheloma cryptatheria* naturally warrants a re-evaluation of the genus *Acheloma* and the morphological and ontogenetic diversity represented therein. Given that ROMVP 88291 is of similar size, maturity, and morphology to other mature *Acheloma* specimens, the quality of certain characters not previously regarded as taxonomically informative can be reinterpreted (e.g. Polley and Reisz [11]) and used to address the previous synonymy of *A*. *dunni* with *A*. *cumminsi*.

One of the most apparent differences noted between *Acheloma dunni* and *A*. *cumminsi* relates to the size of the internarial fontanelle, even though this feature has been largely dismissed in previous studies [11,21]. *A*. *cumminsi* possesses a large embayment along the dorsal midline border between the premaxilla and nasal elements [refer to Dilkes and Reisz [7] Fig 3A], while *A*. *dunni* is distinctive in having an internarial fontanelle that is greatly reduced in size to a tiny opening along the dorsal midline and most importantly restricted to the premaxilla [refer to Polley and Reisz [11] Fig 1A]. This condition is contrary to that of ROMVP 88291 and *A*. *cumminsi*, as well as all trematopids that possess an internarial fontanelle (Table 2). The presence of the internarial fontanelle itself is not unique to Trematopidae as it has independently evolved in other dissorophoid clades as well (e.g. cacopines [31], amphibamids [34], branchiosaurids [35], micromelerpetontids [36]),[37]. It is important to note that the presence of a large internarial fontanelle is typically found in more derived trematopids such as *Acheloma* [1] and is characterized by a distinct morphology of the premaxilla with a recognizable medial wall of the fontanelle. The morphology of this fontanelle does not appear to have been examined closely in other temnospondyls. It is also possible that a reduced internarial fontanelle may be a derived condition of *A*. *dunni*, and a possible autapomorphy of this species. The holotype skulls of *A*. *dunni* OMNH 73281, *A*. *cumminsi* AMNH 4205, and *A*. *cryptatheria* ROMVP 88291 are of similar size, reflecting ontogenetically similar individuals and suggests that these differing morphologies of the internarial fontanelle are taxonomically informative.

Although the LEE in all species of *Acheloma* are greater than that of the LEP, their relative sizes vary considerably, as do the relative elements forming the latter exposures (see Table 2 for complete measurements). In *Acheloma cumminsi* AMNH 4205 [7], the LEE is ~0.6x the diameter of the orbit, whereas in *Acheloma dunni* OMNH 73281 [10], the LEE length and orbit diameter are nearly equal. ROMVP88291 possesses a very large LEE that is ~1.2x larger than the orbit, which contrasts both *A*. *dunni* and *A*. *cumminsi* holotypes. This suggests that the complete size of the LEE, in relation to orbital diameter is likely taxonomically significant and not ontogenetically problematic in this case. Also, in *A*. *dunni*, an anterior portion and secondary lateral expansion of the ectopterygoid form two pieces of the LEE [10], whereas in *A*. *cumminsi*, only one LEE is present [7]. When comparing the sizes in different trematopids possessing both lateral exposures, only *Acheloma* seem to possess an LEE that is larger than the LEP. For example, for both *Phonerpeton pricei* [8] and the juvenile dissorophid *Cacops morrisi* [30] the LEP is about twice the size of the LEE. Interestingly, *A*. *cumminsi* and other trematopids including ROMVP 88291, only preserve one lateral exposure of the ectopterygoid, which suggests that the presence of two distinct LEE ossifications is likely unique to *A*. *dunni*. This distinction appears not to be ontogenetically influenced, as the juvenile specimen *A*. *dunni* (BMRP2007.3.4) also exhibits two pieces of an LEE [10].

The length of the subnarial process of the lacrimal also appears to distinguish *Acheloma dunni* from *A*. *cumminsi* (Table 1). The subnarial processes extending far past the level of the most anterior extension of the prefrontal is considered to be “long”, whereas the processes that are equal to or surpassed by the most anterior extension of the prefrontal are considered to be “short”. The short condition is best exemplified by the short-snouted trematopids (e.g. *Tambachia trogallas* [12], *Fedexia striegeli* [32], *Mordex calliprepes* [1], etc.), with the exceptions of *Ecolsonia cutlerensis* [5] and *Anconastes vesperus* [6], while many of the long-snouted trematopids *Acheloma* [7,10], *Phonerpeton* [8,28], *Rotaryus* [13] retain a “long” subnarial process. This could suggest that the presence of a “long” subnarial process is a derived feature of Trematopidae as they are mostly present in the more derived forms like *Acheloma* and *Ecolsonia cutlerensis* [5]. This would mean that *A*. *dunni* is the only species of long-snouted trematopid to retain this primitive “short” subnarial process. Since juvenile forms (e.g. BMRP2007.3.1 & BMRP2007.3.4 [10]) of *A*. *dunni* also possess this “short” subnarial process, this condition is likely not ontogenetically influenced and can confidently be used as a taxonomically informative character for distinguishing *A*. *dunni* from the other species of *Acheloma*, including *Acheloma cryptatheria*.

The size of the ectopterygoid fangs relative to the marginal teeth has been neglected as a taxonomically informative character even though there is a clear differentiation between that of *Acheloma dunni* and *A*. *cumminsi* for this feature [10]. In *A*. *cumminsi*, the ectopterygoid fangs are about the same size as the largest marginal teeth [7], whereas *A*. *dunni* has fangs that are relatively much larger than any other trematopid and the even largest marginal teeth [10]. Unfortunately, this feature is not clearly observable in many other trematopids due to poor preservation [1,5,13,17]. Since *A*. *dunni*, *A*. *cumminsi*, and *Acheloma cryptatheria* holotypes are all likely mature, differing morphologies of the ectopterygoid fangs are likely not the result of ontogenetic variation and are therefore taxonomically informative.

There are several other morphological differences between *Acheloma dunni* and *A*. *cumminsi* that appear to have been ignored or relegated to variability [21]. *A*. *dunni* displays what is considered the primitive condition of possessing a greater number of small teeth in both premaxillae and maxillae than either *A*. *cumminsi* or *A*. *cryptatheria*, with the latter likely representing the derived condition of possessing fewer, larger marginal teeth [7,10]. Polley and Reisz [11] did not cite this to be taxonomically significant, however the increase in tooth size and simultaneous reduction in marginal tooth count may also be correlated to the difference in the caniniform tooth positioning (Table 1). Dilkes [16] cited the presence of premaxillary and maxillary caniniform teeth to represent the final stage in the postmetamorphic ontogenetic growth of a trematopid skull, which is all displayed by the holotypes of *A*. *dunni*, *A*. *cumminsi*, and *A*. *cryptatheria* ROMVP 88291. This suggests that the position of the caniniform dentition and the marginal tooth count could be confidently used to further distinguish the holotypes of *A*. *dunni* and *A*. *cumminsi* as separate species.

It is notable that there are some important features related to the location of the LEE and LEP as well that can be used to distinguish between *Acheloma* and other trematopids. All species of *Acheloma* possess a lateral exposure of the palatine (LEP) and of the ectopterygoid (LEE), that are excluded from the orbital margin. This is related to an interesting feature of *Acheloma*, the presence of a deep cheek region, and location of the orbit high on the side of the skull. This is reflected by the presence of a deep suborbital flange of the skull roof, one that allows for a strong sutural contact between the jugal and lacrimal bones, excluding the maxilla and the lateral palatal exposure from the orbit.

Given the new evidence provided by the new species *Acheloma cryptatheria* ROMVP 88291 and a re-evaluation of the known *Acheloma* material, we reject the synonymization of *A*. *dunni* and *A*. *cumminsi* and provide a revised diagnosis for both species below. A revised diagnosis for the genus of *Acheloma* is not required as there are no new autapomorphies shared by all members of *Acheloma* that are independent of ontogenetic variation identified in this study.

As such, the revised diagnoses by Gee [21] remains valid and is cited as the following: (1) presence of a dentulous ridge on the vomer extending anteroposteriorly; (2) an exclusion of the lateral exposures of the palatine (LEP) and of the ectopterygoid (LEE) from the orbit by a lacrimal-jugal contact, although this may be only reliably found in adults. Although not autapomorphies, Gee further differentiates *Acheloma* from *Ecolsonia cutlerensis*, *Fedexia striegeli* and *Mattauschia laticeps* by (3) presence of an internarial fontanelle; (4) presence of an elongated snout where the naris is significantly larger than the orbit; (5) a dorsoventrally constricted otic notch with a reduced lateral exposure of the supratympanic flange; and (6) the anteromedial expansion of the choana, forming a ‘Y’-shaped contour.

A revised diagnosis for *Acheloma dunni* (OMNH 73281) is presented below:

    **Temnospondyli Zittel, 1888**

    **Dissorophoidea Bolt, 1969**

    **Xerodromes Schoch and Milner, 2014**

    **Olsoniformes Anderson et al., 2008**

    **Trematopidae Williston, 1910**

    ***Acheloma* Cope, 1882**

    ***Acheloma dunni* Polley and Reisz, 2011**

### Revised diagnosis

A trematopid temnospondyl amphibian that can be distinguished from other species of the genus *Acheloma* by the following autapomorphic feature: presence of a reduced internarial fontanelle and confined to the premaxilla.

Differs from other members of the genus in the presence of two distinct ossifications of the lateral exposure of the ectopterygoid in the adults, which are collectively equal to the diameter of the orbit; presence of a short subnarial process that is at level of or surpassed by the most anterior extension of the prefrontal; premaxilla with space for about thirteen marginal teeth; maxillary element with space for approximately twenty eight marginal teeth.

A revised diagnosis for *Acheloma cumminsi* (AMNH 4205) is presented below:

    **Temnospondyli Zittel, 1888**

    **Dissorophoidea Bolt, 1969**

    **Xerodromes Schoch and Milner, 2014**

    **Olsoniformes Anderson et al., 2008**

    **Trematopidae Williston, 1910**

    ***Acheloma* Cope, 1882**

    ***Acheloma cumminsi* Cope, 1882**

### Revised diagnosis

Same as for genus as provided by Gee [21] but differs from *Acheloma dunni* in addition to the following characters: presence of an enlarged internarial fontanelle bordering the premaxilla and nasal contact; presence of a long subnarial process that extends past the most anterior extension of the prefrontal; premaxilla with space for approximately nine marginal teeth; maxillary element with space for approximately twenty three to twenty six marginal teeth.

Differs from *Acheloma cryptatheria* in the presence of ectopterygoid fangs approximately the same size as largest marginal teeth; lateral exposure of the ectopterygoid smaller than the diameter of the orbit.

At this time, due to uncertainty regarding the quality of preservation of the external surface of the snout, which suggests that *A*. *cumminsi* preserves a combination of reticulate and ride-and-groove pattern of ornamentation, we refrain from using this feature in our differential diagnosis.

## Conclusion

The available evidence clearly shows that two distinct species of *Acheloma* are represented at the Richards Spur locality, distinguished by the pattern of the suborbital lateral exposure of the palatal elements, the distinct size of the palatal fangs and anterior marginal dentition, the pattern of ornamentation, and size of the internarial fontanelle. These differences suggest that the new species appears to have a more robust cranium, with some differences in its feeding strategy from the other valid species at this locality, *Acheloma dunni*. Interestingly, there are numerous other specimens of trematopids at this locality, mostly the remains of smaller, potentially juvenile individuals. These new specimens, and the juvenile individuals that were included in previous studies of the trematopid fauna of this locality may help clarify the current controversies related to the ontogeny of trematopids, which complicates the use of certain characters for confidently diagnosing and evaluating evolutionary relationships among members of this clade. However, this is beyond the scope of this paper and will instead be explored in future studies.
